# Search for neutrinos from decaying dark matter with IceCube: IceCube Collaboration

**DOI:** 10.1140/epjc/s10052-018-6273-3

**Published:** 2018-10-16

**Authors:** M. G. Aartsen, M. Ackermann, J. Adams, J. A. Aguilar, M. Ahlers, M. Ahrens, I. Al Samarai, D. Altmann, K. Andeen, T. Anderson, I. Ansseau, G. Anton, C. Argüelles, J. Auffenberg, S. Axani, P. Backes, H. Bagherpour, X. Bai, J. P. Barron, S. W. Barwick, V. Baum, R. Bay, J. J. Beatty, J. Becker Tjus, K.-H. Becker, S. BenZvi, D. Berley, E. Bernardini, D. Z. Besson, G. Binder, D. Bindig, E. Blaufuss, S. Blot, C. Bohm, M. Börner, F. Bos, S. Böser, O. Botner, E. Bourbeau, J. Bourbeau, F. Bradascio, J. Braun, M. Brenzke, H.-P. Bretz, S. Bron, J. Brostean-Kaiser, A. Burgman, R. S. Busse, T. Carver, E. Cheung, D. Chirkin, A. Christov, K. Clark, L. Classen, G. H. Collin, J. M. Conrad, P. Coppin, P. Correa, D. F. Cowen, R. Cross, P. Dave, M. Day, J. P. A. M. de André, C. De Clercq, J. J. DeLaunay, H. Dembinski, S. De Ridder, P. Desiati, K. D. de Vries, G. de Wasseige, M. de With, T. DeYoung, J. C. Díaz-Vélez, V. di Lorenzo, H. Dujmovic, J. P. Dumm, M. Dunkman, E. Dvorak, B. Eberhardt, T. Ehrhardt, B. Eichmann, P. Eller, P. A. Evenson, S. Fahey, A. R. Fazely, J. Felde, K. Filimonov, C. Finley, S. Flis, A. Franckowiak, E. Friedman, A. Fritz, T. K. Gaisser, J. Gallagher, E. Ganster, L. Gerhardt, K. Ghorbani, W. Giang, T. Glauch, T. Glüsenkamp, A. Goldschmidt, J. G. Gonzalez, D. Grant, Z. Griffith, C. Haack, A. Hallgren, L. Halve, F. Halzen, K. Hanson, D. Hebecker, D. Heereman, K. Helbing, R. Hellauer, S. Hickford, J. Hignight, G. C. Hill, K. D. Hoffman, R. Hoffmann, T. Hoinka, B. Hokanson-Fasig, K. Hoshina, F. Huang, M. Huber, K. Hultqvist, M. Hünnefeld, R. Hussain, S. In, N. Iovine, A. Ishihara, E. Jacobi, G. S. Japaridze, M. Jeong, K. Jero, B. J. P. Jones, P. Kalaczynski, W. Kang, A. Kappes, D. Kappesser, T. Karg, A. Karle, U. Katz, M. Kauer, A. Keivani, J. L. Kelley, A. Kheirandish, J. Kim, M. Kim, T. Kintscher, J. Kiryluk, T. Kittler, S. R. Klein, R. Koirala, H. Kolanoski, L. Köpke, C. Kopper, S. Kopper, J. P. Koschinsky, D. J. Koskinen, M. Kowalski, K. Krings, M. Kroll, G. Krückl, S. Kunwar, N. Kurahashi, T. Kuwabara, A. Kyriacou, M. Labare, J. L. Lanfranchi, M. J. Larson, F. Lauber, K. Leonard, M. Lesiak-Bzdak, M. Leuermann, Q. R. Liu, E. Lohfink, C. J. Lozano Mariscal, L. Lu, J. Lünemann, W. Luszczak, J. Madsen, G. Maggi, K. B. M. Mahn, S. Mancina, R. Maruyama, K. Mase, R. Maunu, K. Meagher, M. Medici, M. Meier, T. Menne, G. Merino, T. Meures, S. Miarecki, J. Micallef, G. Momenté, T. Montaruli, R. W. Moore, M. Moulai, R. Nahnhauer, P. Nakarmi, U. Naumann, G. Neer, H. Niederhausen, S. C. Nowicki, D. R. Nygren, A. Obertacke Pollmann, A. Olivas, A. O’Murchadha, E. O’Sullivan, T. Palczewski, H. Pandya, D. V. Pankova, P. Peiffer, J. A. Pepper, C. Pérez de los Heros, D. Pieloth, E. Pinat, M. Plum, P. B. Price, G. T. Przybylski, C. Raab, L. Rädel, M. Rameez, L. Rauch, K. Rawlins, I. C. Rea, R. Reimann, B. Relethford, M. Relich, E. Resconi, W. Rhode, M. Richman, S. Robertson, M. Rongen, C. Rott, T. Ruhe, D. Ryckbosch, D. Rysewyk, I. Safa, S. E. Sanchez Herrera, A. Sandrock, J. Sandroos, M. Santander, S. Sarkar, S. Sarkar, K. Satalecka, M. Schaufel, P. Schlunder, T. Schmidt, A. Schneider, S. Schoenen, S. Schöneberg, L. Schumacher, S. Sclafani, D. Seckel, S. Seunarine, J. Soedingrekso, D. Soldin, M. Song, G. M. Spiczak, C. Spiering, J. Stachurska, M. Stamatikos, T. Stanev, A. Stasik, R. Stein, J. Stettner, A. Steuer, T. Stezelberger, R. G. Stokstad, A. Stößl, N. L. Strotjohann, T. Stuttard, G. W. Sullivan, M. Sutherland, I. Taboada, J. Tatar, F. Tenholt, S. Ter-Antonyan, A. Terliuk, S. Tilav, P. A. Toale, M. N. Tobin, C. Tönnis, S. Toscano, D. Tosi, M. Tselengidou, C. F. Tung, A. Turcati, C. F. Turley, B. Ty, E. Unger, M. Usner, J. Vandenbroucke, W. Van Driessche, D. van Eijk, N. van Eijndhoven, S. Vanheule, J. van Santen, M. Vraeghe, C. Walck, A. Wallace, M. Wallraff, F. D. Wandler, N. Wandkowsky, A. Waza, C. Weaver, M. J. Weiss, C. Wendt, J. Werthebach, S. Westerhoff, B. J. Whelan, K. Wiebe, C. H. Wiebusch, L. Wille, D. R. Williams, L. Wills, M. Wolf, J. Wood, T. R. Wood, E. Woolsey, K. Woschnagg, G. Wrede, D. L. Xu, X. W. Xu, Y. Xu, J. P. Yanez, G. Yodh, S. Yoshida, T. Yuan

**Affiliations:** 1 III. Physikalisches Institut, RWTH Aachen University, 52056 Aachen, Germany; 2 Department of Physics, University of Adelaide, Adelaide, 5005 Australia; 3 Department of Physics and Astronomy, University of Alaska Anchorage, 3211 Providence Dr., Anchorage, AK 99508 USA; 4 Department of Physics, University of Texas at Arlington, 502 Yates St., Science Hall Rm 108, Box 19059, Arlington, TX 76019 USA; 5 CTSPS, Clark-Atlanta University, Atlanta, GA 30314 USA; 6 School of Physics and Center for Relativistic Astrophysics, Georgia Institute of Technology, Atlanta, GA 30332 USA; 7 Department of Physics, Southern University, Baton Rouge, LA 70813 USA; 8 Department of Physics, University of California, Berkeley, CA 94720 USA; 9 Lawrence Berkeley National Laboratory, Berkeley, CA 94720 USA; 10 Institut für Physik, Humboldt-Universität zu Berlin, 12489 Berlin, Germany; 11 Fakultät für Physik und Astronomie, Ruhr-Universität Bochum, 44780 Bochum, Germany; 12 Science Faculty CP230, Université Libre de Bruxelles, 1050 Brussels, Belgium; 13 Dienst ELEM, Vrije Universiteit Brussel (VUB), 1050 Brussels, Belgium; 14 Department of Physics, Massachusetts Institute of Technology, Cambridge, MA 02139 USA; 15 Department of Physics and Institute for Global Prominent Research, Chiba University, Chiba, 263-8522 Japan; 16 Department of Physics and Astronomy, University of Canterbury, Private Bag 4800, Christchurch, New Zealand; 17 Department of Physics, University of Maryland, College Park, MD 20742 USA; 18 Department of Physics and Center for Cosmology and Astro-Particle Physics, Ohio State University, Columbus, OH 43210 USA; 19 Department of Astronomy, Ohio State University, Columbus, OH 43210 USA; 20 Niels Bohr Institute, University of Copenhagen, 2100 Copenhagen, Denmark; 21 Department of Physics, TU Dortmund University, 44221 Dortmund, Germany; 22 Department of Physics and Astronomy, Michigan State University, East Lansing, MI 48824 USA; 23 Department of Physics, University of Alberta, Edmonton, AB T6G 2E1 Canada; 24 Erlangen Centre for Astroparticle Physics, Friedrich-Alexander-Universität Erlangen-Nürnberg, 91058 Erlangen, Germany; 25 Département de Physique Nucléaire et Corpusculaire, Université de Genève, 1211 Geneva, Switzerland; 26 Department of Physics and Astronomy, University of Gent, 9000 Ghent, Belgium; 27 Department of Physics and Astronomy, University of California, Irvine, CA 92697 USA; 28 Department of Physics and Astronomy, University of Kansas, Lawrence, KS 66045 USA; 29 SNOLAB, 1039 Regional Road 24, Creighton Mine 9, Lively, ON P3Y 1N2 Canada; 30 Department of Astronomy, University of Wisconsin, Madison, WI 53706 USA; 31 Department of Physics and Wisconsin IceCube Particle Astrophysics Center, University of Wisconsin, Madison, WI 53706 USA; 32 Institute of Physics, University of Mainz, Staudinger Weg 7, 55099 Mainz, Germany; 33 Department of Physics, Marquette University, Milwaukee, WI 53201 USA; 34 Physik-Department, Technische Universität München, 85748 Garching, Germany; 35 Institut für Kernphysik, Westfälische Wilhelms-Universität Münster, 48149 Münster, Germany; 36 Department of Physics and Astronomy, Bartol Research Institute, University of Delaware, Newark, DE 19716 USA; 37 Department of Physics, Yale University, New Haven, CT 06520 USA; 38 Department of Physics, University of Oxford, 1 Keble Road, Oxford, OX1 3NP UK; 39 Department of Physics, Drexel University, 3141 Chestnut Street, Philadelphia, PA 19104 USA; 40 Physics Department, South Dakota School of Mines and Technology, Rapid City, SD 57701 USA; 41 Department of Physics, University of Wisconsin, River Falls, WI 54022 USA; 42 Department of Physics and Astronomy, University of Rochester, Rochester, NY 14627 USA; 43 Department of Physics, Oskar Klein Centre, Stockholm University, 10691 Stockholm, Sweden; 44 Department of Physics and Astronomy, Stony Brook University, Stony Brook, NY 11794-3800 USA; 45 Department of Physics, Sungkyunkwan University, Suwon, 440-746 Korea; 46 Department of Physics and Astronomy, University of Alabama, Tuscaloosa, AL 35487 USA; 47 Department of Astronomy and Astrophysics, Pennsylvania State University, University Park, PA 16802 USA; 48 Department of Physics, Pennsylvania State University, University Park, PA 16802 USA; 49 Department of Physics and Astronomy, Uppsala University, Box 516, 75120 Uppsala, Sweden; 50 Department of Physics, University of Wuppertal, 42119 Wuppertal, Germany; 51 DESY, 15738 Zeuthen, Germany

## Abstract

With the observation of high-energy astrophysical neutrinos by the IceCube Neutrino Observatory, interest has risen in models of PeV-mass decaying dark matter particles to explain the observed flux. We present two dedicated experimental analyses to test this hypothesis. One analysis uses 6 years of IceCube data focusing on muon neutrino ‘track’ events from the Northern Hemisphere, while the second analysis uses 2 years of ‘cascade’ events from the full sky. Known background components and the hypothetical flux from unstable dark matter are fitted to the experimental data. Since no significant excess is observed in either analysis, lower limits on the lifetime of dark matter particles are derived: we obtain the strongest constraint to date, excluding lifetimes shorter than $10^{28}\hbox { s}$ at 90% CL for dark matter masses above $10~\hbox {TeV}$.

## High-energy neutrinos and dark matter decay

To this day, the origin of the flux of high-energy neutrinos discovered by IceCube [1, 2] remains unidentified [3]. Likewise, the nature and properties of dark matter (DM) are among the most important open questions in physics. If the hypothetical dark matter particles are unstable on time-scales longer than the age of the universe, then the two questions may be linked [4, 5], i.e. neutrinos produced in dark matter decays could contribute to the observed astrophysical flux. Following the IceCube discovery of cosmic neutrinos up to $\hbox {Pev}$ energies, there has been renewed interest in this possibility [6–20]. In particular, the connection between neutrinos and gamma-rays from DM decay has been discussed in further detail [21–32].

We present two dedicated analyses to test whether the description of the observed neutrino flux can be improved by an additional component from heavy ($m_{\text {DM}} > 10~\hbox {TeV}$) dark matter decays as an alternative to bottom-up scenarios of astrophysical acceleration [33]. Such heavy particles are receiving increased attention because the classic WIMP paradigm of weak-scale mass dark matter is disfavoured by the negative results in searches for new physics at the LHC [34], in direct DM detection experiments [35–39], and in searches for DM annihilation into neutrinos [40, 41] or gamma-rays [42–46].

Our results significantly improve upon the best previous experimental bounds on decaying dark matter obtained with gamma rays [44–47], neutrinos [48], and those derived from high-energy cosmic rays and the cosmic microwave background radiation [4, 5].

## IceCube detector and event selections

IceCube is a cubic-kilometer ice Cherenkov detector located at the South Pole, situated between 1450 and 2450 m below the surface [49]. Charged particles produced in neutrino interactions with the Antarctic ice or the bedrock below are detected by the Cherenkov light they emit, allowing the reconstruction of the originating neutrino’s direction and energy [50].

The presented analyses use two different event samples. The first analysis is based on 6 years of $\nu _{\mu }$ charged-current data collected between 2009 and 2015, i.e., track-like events from the Northern Hemisphere. More details can be found in Ref. [2]. The second analysis uses 2 years of data collected between 2010 and 2012. The event selection is based on a previous study [51], modified to select only cascade events from the full sky which are produced in NC interactions or CC interactions of $\nu _{e}$ or $\nu _{\tau }$. Note that in the following no distinction is made between particles and anti-particles; the labels *neutrino* and *lepton* include the respective anti-particles and the used cross-sections incorporate both particles and anti-particles.

The two analysis samples are statistically independent, and while the track sample contains a much larger number of events, the full-sky coverage and better energy resolution of the cascade sample (see Table 1) lead to comparable sensitivities.

**Table 1:** Summary of the two event samples. Detailed sample descriptions can be found in Refs. [2, 51]

	Tracks	Cascades
Number of events	352,294	278
Livetime	2060 days	641 days
Sky coverage	North ($\text {zenith}>85^\circ$)	Full Sky
Atm. muon background	0.3%	10%
Median reconstr. error	$<0.5^{\circ } (\text {E}_{\nu }>100~\hbox {TeV})$	$\sim 10^{\circ }$
Energy uncertainty	$\sim 100\%$	$\sim 10\%$

## Analysis

To test whether the observed flux of high-energy neutrinos (partly) arises from heavy decaying dark matter, a forward-folding likelihood fit of the distribution of reconstructed energy and direction is performed on both datasets, similar to Refs. [2, 51]. The total observed flux is modelled as a sum of background and signal flux components. Each of these components is described by a parametrized flux template that depends on the fitted model parameters.

### Flux components

Cosmic-ray air showers produce secondary mesons which decay into charged leptons and neutrinos. These atmospheric neutrinos are the main source of background in both data samples. They can be further divided into *conventional* atmospheric neutrinos produced by the decay of pions and kaons and *prompt* neutrinos produced by the decay of charmed mesons. This latter flux is sub-dominant at high energies and has not been separately identified yet [2]. Atmospheric neutrino flux predictions are taken from Refs. [52, 53] for the conventional (modified to account for the cosmic-ray knee [2]) and prompt component, respectively. From the Southern Hemisphere, cosmic-ray induced atmospheric muons can also penetrate the ice, reach the detector and mimic a neutrino signal. After application of appropriate event selections, the atmospheric muon contamination is negligible in the track-like sample and below 10% in the cascade sample.

Astrophysical neutrinos from cosmic rays interacting in or near their production sites constitute a second background flux to the targeted signal of neutrinos from decaying dark matter. Since the origin of cosmic rays is unknown, an exact modelling of this astrophysical flux is not possible. A generic parametrization of these astrophysical neutrinos as an isotropic flux with a power–law energy spectrum agrees well with present measurements [1, 2] and is therefore used in the fitting. The spectral index $\gamma$ and the flux normalization $\varPhi _\text {astro}$ are taken as free parameters in the fit.

**Fig. 1:**
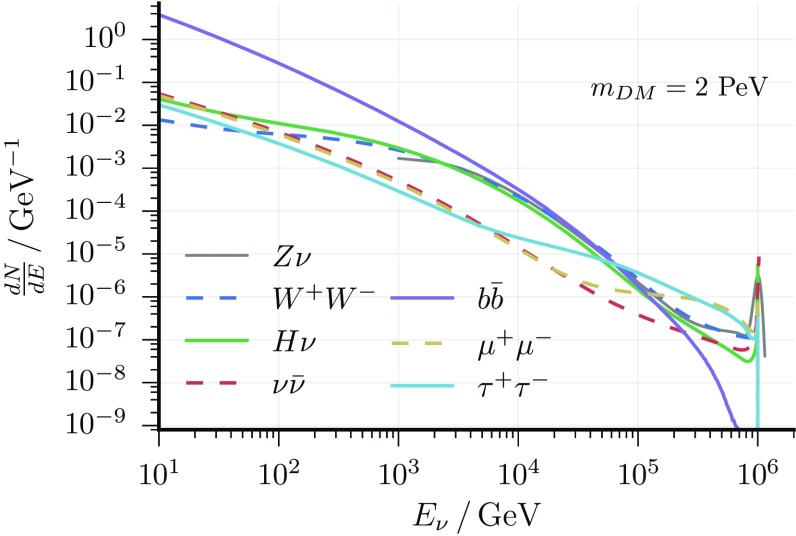
Neutrino yield per decay as a function of neutrino energy (flavour-averaged): all considered decay channels ($\text {BR}=100\%$) are presented for an assumed dark matter mass of $2~\hbox {PeV}$

When heavy dark matter decays into standard model particles, neutrinos are necessarily expected in the final state [54]. Observing these neutrinos would thus constitute an indirect probe of the scenario of decaying dark matter. The energy spectrum, $dN_{\nu }/dE_{\nu }$, of the expected neutrinos depends on the exact decay mechanism and is model dependent. In this analysis, several “hard” (e.g., dark matter decaying directly into neutrinos [8, 55, 56]) and “soft” (e.g., neutrinos produced in the subsequent hadronic decay-chain of standard model particles [6]) decays are used as benchmark channels. Their spectra were simulated with PYTHIA 8.1 [57] and are shown in Fig. 1.

**Fig. 2:**
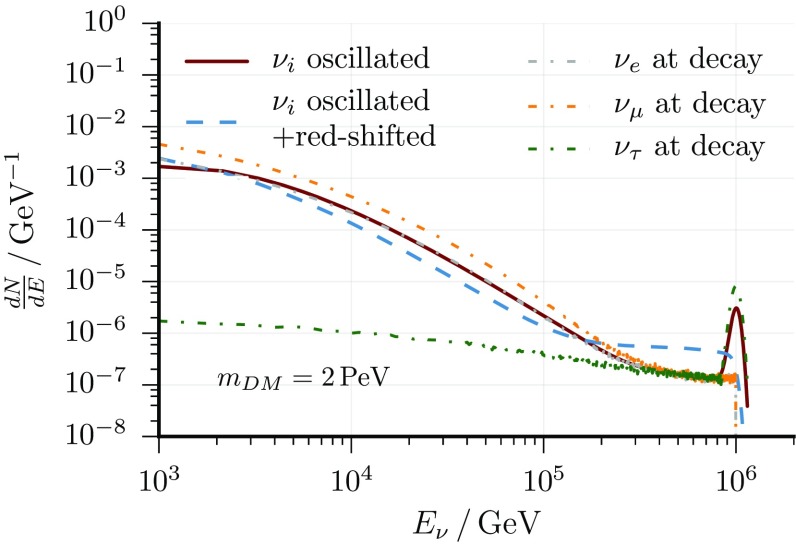
Neutrino yield per decay as a function of neutrino energy assuming the hard decay channel $DM\rightarrow Z+\nu _{\tau }$: the effects of neutrino mixing and red-shift are illustrated

At Earth, the neutrino flux from dark matter decays has to be subdivided into a galactic and an extragalactic component. The expected energy distribution of the galactic component $\varPhi ^{\text {Halo}}$ follows the initial decay spectrum. Its angular distribution incorporates the (uncertain) distribution of dark matter in the Milky Way halo via the line-of-sight integral [58]. The Burkert halo profile [59, 60] with best-fit parameters from Ref. [61] is used as a benchmark and other halo profiles are considered as systematic uncertainties. The extragalactic neutrino flux from dark matter $\varPhi ^{\text {Cosm.}}$ is expected to be isotropic and to have a red-shifted decay spectrum in energy. This flux is calculated adopting the $\varLambda$CDM cosmological model with parameters from Ref. [62]. The total signal flux is computed as the sum of both fluxes assuming that a single dark matter particle constitutes the observed dark matter in the universe. Additionally, neutrino mixing is applied with parameters from Ref. [63], the effects are shown in Fig. 2. The total flux depends on two fit parameters: the mass $m_{\text {DM}}$, which determines the energy cut-off, and the lifetime $\tau _{\text {DM}}$ of the dark matter particle, which determines the normalization. Explicitly, it is given by

1 $$\begin{aligned} \frac{d\varPhi }{dE_{\nu }}= & {} \frac{1}{4\pi m_{\text {DM}} \tau _{\text {DM}}} \left( \frac{d\varPhi ^{\text {Halo}}}{dE_{\nu }} +\frac{d\varPhi ^{\text {Cos.}}}{dE_{\nu }} \right) ,\nonumber \\ \frac{d\varPhi ^{\text {Halo}}}{dE_{\nu }}= & {} \frac{dN_{\nu }}{dE_{\nu }} \int \limits _0^{\infty } \rho _{\text {DM}}(r(s)) \, ds , \nonumber \\ \frac{d\varPhi ^{\text {Cos.}}}{dE_{\nu }}= & {} \frac{\varOmega _{\text {DM}} \rho _{\text {c}}}{H_0} \int \limits _0^{\infty } \frac{dN_{\nu }}{d(E_{\nu }(1+z))}\frac{dz}{\sqrt{\varOmega _{\varLambda }+\varOmega _{\text {m}}(1+z)^3}}. \end{aligned}$$

### Likelihood analysis

In order to find the combination of the flux components that describe the data best, a forward-folding likelihood fit is performed. Flux templates, as a function of the fit parameters, are generated from a dedicated simulation of the detector response (see Refs. [2, 51] for more details) and then compared to the observed event distributions in reconstructed energy *E*, right-ascension $\alpha$, and zenith angle $\theta$. Given a set of observed events, *N*, and the predicted number of events, $\mu _i(\xi )$, the Poisson likelihood is calculated and the fit parameters $\xi$ are optimized, namely,

2 $$\begin{aligned} L ( N ; \xi ) =\prod _{i=1}^{\text {bins}} \text {P}_{\text {Poisson}}(n_{i};\mu _{i}(E_{j},\alpha _{j},\theta _{j}; \xi )). \end{aligned}$$

While a binned likelihood method is used in the analysis of the track-like events, an unbinned approach is used in the analysis of the cascade sample, which corresponds to the limit of infinitesimal bin size.

To quantify the statistical significance of the best fit result, a test statistic is defined as the ratio of the maximum likelihood values for the background-only case (atmospheric and astrophysical fluxes) and for the background-plus-signal case (i.e., including the additional flux from dark matter decay), namely:

3 $$\begin{aligned} \text {TS}:= 2 \times \log \left( \frac{L(\hat{\phi }_{\text {atm.}}, \hat{\phi }_{\text {astro}},\hat{\gamma },\hat{m_{\text {DM}}},\hat{\tau _{\text {DM}}})}{L(\hat{\hat{\phi }}_{\text {atm.}},\hat{\hat{\phi }}_{\text {astro}},{\hat{\hat{\gamma }}},{\tau _{\text {DM}}}=\infty )}\right) \ge 0. \end{aligned}$$

Since the signal-plus-background case has additional degrees of freedom (four vs. two physical fit parameters), the TS value will always be positive. The observed TS value of the best-fit result is then compared to pseudo-experiments of the background and different signal hypotheses to construct confidence intervals.

### Systematics

The systematic uncertainties of the two analyses arise from the modelling of the dark matter halo, the detector and the background fluxes. The dominant systematic uncertainty is the poorly understood dark matter distribution in our galactic halo. To investigate the resulting effect, the Burkert halo parameters are varied within intervals of one standard deviation while keeping their correlation fixed, by selecting $\beta _2=-0.5$ (see discussion in Ref. [61]). In addition, the impact of a different halo profile, namely the Navarro–Frenk–White [64, 65] profile, with best-fit parameters from Ref. [61], on the fit results is studied. The total effect of these halo model variations on the derived lifetime limit is $\pm \,10\%$. This value is consistent across all the masses and decay channels and between the two analyses. The uncertainty on the extragalactic flux component, which arises from the average extragalactic dark matter density, is on the order of a few percent [62] and is thus not considered here.

Detector simulation and background flux uncertainties are treated differently between the two analyses. In the analysis of track-like events, several nuisance parameters are fitted simultaneously in order to absorb deviations from the baseline expectation (see Ref. [2] for more details). They include the normalization of the prompt atmospheric flux, cosmic-ray flux model uncertainties, relative contribution from pion and kaon decays to the atmospheric fluxes, optical properties of the glacial ice, and the optical efficiency of the detector.

In the analysis of the cascade-like events, prompt atmospheric flux uncertainties [51], errors in the event reconstruction due to ice model uncertainties [66], a 10% uncertainty on the optical efficiency of the detector, and the impact of the finite simulation statistics are taken into account. The data are reanalyzed under different assumptions within the systematic uncertainties and the spread of the resulting limits is taken as the overall systematic uncertainty.

## Results

### Fit results

To address the question of whether the observed flux of cosmic neutrinos can be described significantly better by including a component from decaying dark matter, the hard decay channels $DM \rightarrow H + \nu$ (cascades) and $DM \rightarrow Z + \nu$ (tracks) are fitted to the respective data. A dark matter signal would be expected to show up in both analyses. Also note, that the observable energy distributions are smeared out due to the limited detector resolution and the cosmological red-shift. It is therefore sufficient to fit these single decay channels in order to test whether a contribution from dark matter is present and multiple tests are not necessary. The obtained best-fit results and the corresponding p-values with respect to the background only hypothesis are listed in Table 2. The fits of the background-only hypothesis agree well with the results in Refs. [2, 51]. Small differences arise due to a different choice of bins (tracks) and the altered selection (cascades).

**Table 2:** Best-fit results assuming the decay channels $DM \rightarrow H + \nu$ (cascades) and $DM \rightarrow Z + \nu$ (tracks). Background p-values are stated in brackets

	Tracks		Cascades	
	Bg.	Signal+Bg.	Bg.	Signal+Bg.
$m_{\text {DM}}\,/\,\text {PeV}$	–	1.3	–	0.1
$\tau _{\text {DM}}\,/\,10^{27}\text {s}$	–	22	–	8.3
$\hbox {Astroph. norm.}^{\mathrm{a}}$	0.97	0.16	2.15	1.62
Spectr. index	2.16	1.99	2.75	2.81
$\text {TS} = 2\times \varDelta \text {LLH}$	$6.7\,(p=0.035$)		$3.4\,(p=0.55)$	

$^{\mathrm{a}}$Normalization in units of $10^{-18}\,\hbox {GeV}^{-1}\,\hbox {cm}^{-2}\,\hbox {sr}^{-1}\,\hbox {s}^{-1}$

**Fig. 3:**
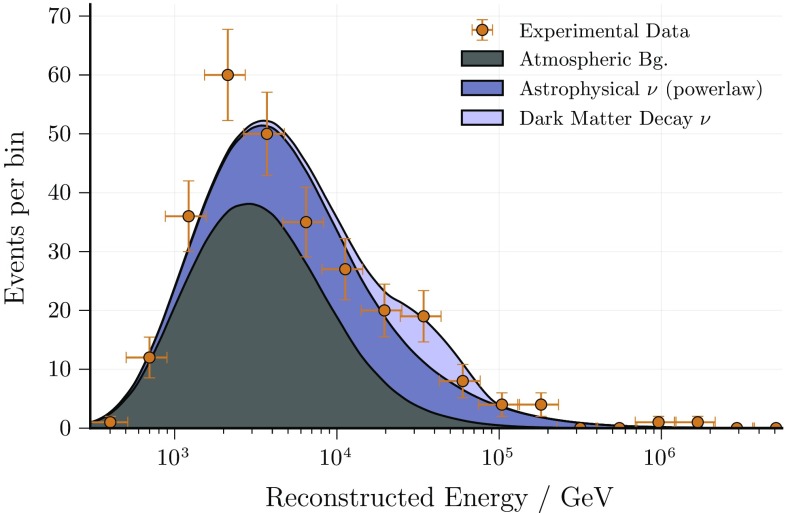
Cascade analysis: best-fit energy distribution for the signal hypothesis (components stacked to illustrate the dark matter component), with the best fit parameters listed in Table 2. The fit is performed on un-binned data, but for visualization purposes a binning is applied in the figure

**Fig. 4:**
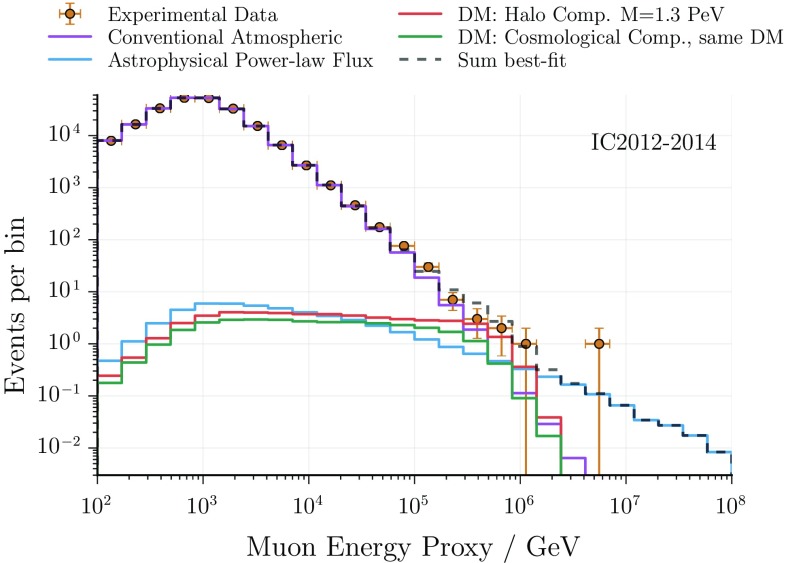
Track analysis: best-fit energy distribution. While the low-energy events are well described by the conventional atmospheric component, the high-energy events are modelled by a combination of a weak diffuse astrophysical flux and a component from decaying dark matter (best-fit parameters in Table 2). The figure shows data recorded between 2012 and 2014 as they are based on the same energy estimator (see [67] for more details). The remaining years are fitted simultaneously but are not shown here

The corresponding best-fit distributions in reconstructed energy are shown in Figs. 3 and 4 together with the experimental data. Note that different energy estimators are used in the sub-samples (data-taking seasons) of the track analysis [67]. It is therefore not possible to show the experimental data in one histogram.

### Interpretation of the fit results

**Fig. 5:**
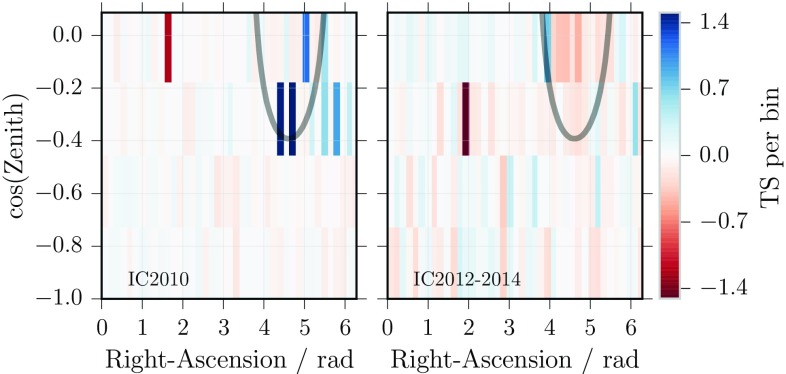
Track analysis: TS per bin to illustrate the time-dependency of the fit result: blue bins show agreement with the signal hypothesis, red bins favour a purely diffuse astrophysical flux. The gray line indicates the direction where most of the dark matter signal is expected (line-of-sight integral at half of the central value)

Although the best-fit result in both analyses includes a non-zero dark matter component, the results are not significant (as both p-values are above $1\%$). More degrees of freedom in the modelling of the astrophysical flux, e.g. adding a second component, would further reduce the significance. Thus, the result is not interpreted as a signal of dark matter decay. Furthermore, a dark matter signal should be constant in time but the fit of the track-like events shows fluctuations; see Fig. 5: while those bins contributing most strongly in the fit to the data from the first 3 years (e.g., 2010) coincide with the approximate direction of the dark matter halo, such a correlation is disfavoured by the data from 2012 to 2014.

**Fig. 6:**
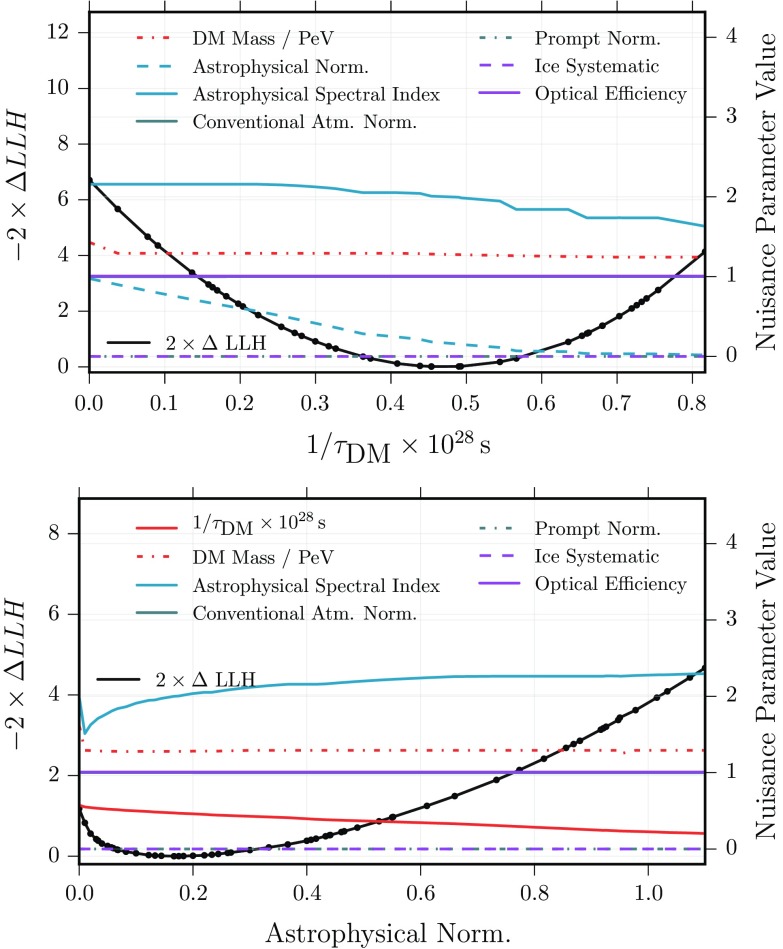
Track analysis: profile likelihood scans of the inverse dark matter lifetime (proportional to the signal strength) and the diffuse astrophysical flux normalization in units of $10^{-18}\hbox {GeV}^{-1}\hbox {cm}^{-2}\hbox {sr}^{-1}\hbox {s}^{-1}$

Another interesting observation is the interplay of the diffuse astrophysical flux and the dark matter component in the fit of track-like events: Fig. 6 shows the profile likelihood of the respective normalizations together with the fit result of other selected parameters. The best-fit astrophysical normalization is significantly reduced compared to previous results [2]. A dark matter only scenario, where the normalization of the astrophysical flux is zero, is however disfavoured by $2\varDelta LLH\simeq 1$ compared to the best-fit point. As expected, the best-fit dark matter mass that induces a cut-off in the energy spectrum is found to be independent of the diffuse astrophysical normalization while the dark matter normalization is anti-correlated.

### Lifetime limits

**Fig. 7:**
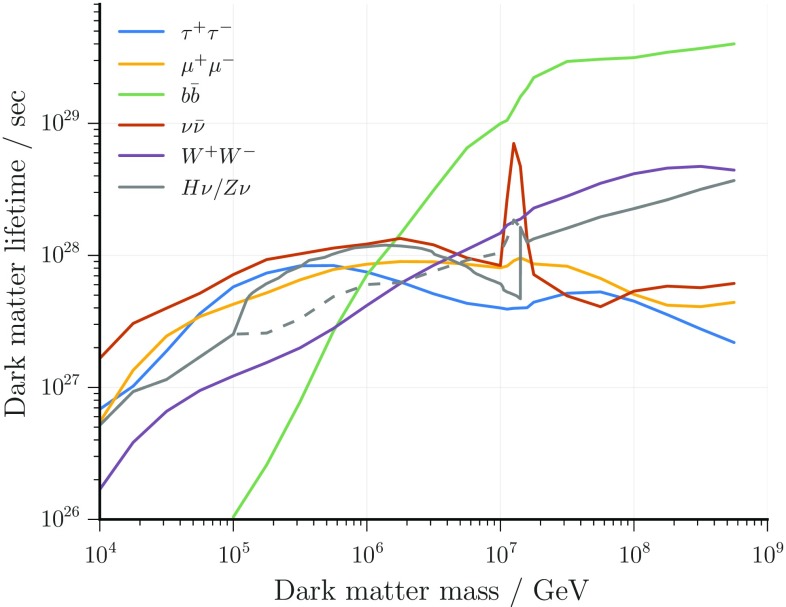
Dark matter lifetime limits for all considered decay channels. For the $Z + \nu / H + \nu$ channel, the limit was combined (solid grey line) as described in the text. Between $m_{\text {DM}}\sim 10^5\,\hbox {GeV}$ and $m_{\text {DM}}=1.5\times 10^7\,\hbox {GeV}$ the limit is obtained from the more sensitive track analysis. The limit from the cascade analysis is shown as a dashed line and turns out to be stronger above $m_{\text {DM}}\sim 5 \times 10^{7}~\hbox {GeV}$

Since no significant dark matter signal is observed, lower limits on the lifetime of the dark matter particle (corresponding to upper limits on its signal strength) are derived. In order to combine the two analyses, the lower limit on the lifetime is extracted from the respective analysis with the better sensitivity (median limit obtained from background pseudo-experiments) at each dark matter mass. The hard decay channels $\mathrm{Z}+\nu$ (track analysis) and $\mathrm{H}+\nu$ (cascade analysis) are treated as the same channel because the resulting neutrino spectra are indistinguishable within energy resolutions. Further, limits for the decay channels $\nu \bar{\nu }$, $\tau ^{+}\tau ^{-}$, $\mu ^{+}\mu ^{-}$, $W^{+}W^{-}$ and $b\bar{b}$ are calculated only in the cascade analysis because the energy resolution of the track analysis is not sufficient to differentiate those channels from each other. The resulting lower limits on the dark matter lifetime are shown in Fig. 7. Note that for the $b\bar{b}$ decay channel, the lower limit on the lifetime increases steeply with the dark matter mass because QCD fragmentation generates a soft tail of low-energy neutrinos (see Fig. 1) which become increasingly relevant for large dark matter mass. Furthermore, no limit on the lifetime is calculated in this channel for $m_{\text {DM}}$ below $10^5\,\,\hbox {GeV}$ because the resulting decay spectrum becomes similar to the atmospheric background fluxes and the respective uncertainties would have a major effect on the obtained limit. The enhanced limits at $m_{\text {DM}}\sim 10^7~\hbox {GeV}$, correspond to the non-observation of electron neutrino events from the expected Glashow resonance [68]. For the track-like sample, all nuisance parameters are fitted to their expectation values within one standard deviation, and the effect on the signal hypothesis is found to be negligible. For the cascade-like sample, the overall impact of the systematics is roughly 10–15% for dark matter masses below 5 PeV and 1% for those above it. The limits shown here include a degradation due to $1\sigma$ systematic variation.

**Fig. 8:**
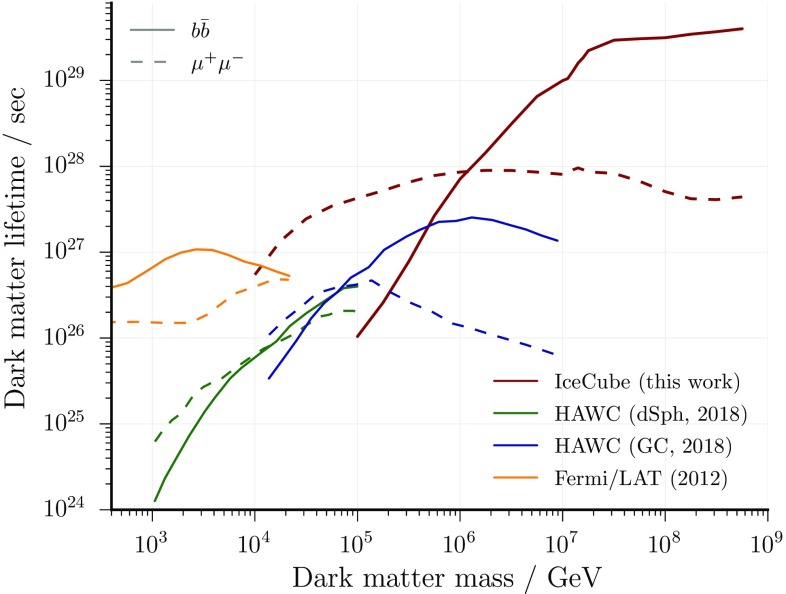
Comparison of the lower lifetime limits with results obtained from gamma-ray telescopes: HAWC (Dwarf Spheroidal Galaxies) [44], HAWC (Galactic Halo/Center) [45] and Fermi/LAT [47]

## Conclusions

Two analyses on statistically independent datasets searching for a contribution from decaying dark matter to the astrophysical neutrino flux have been presented. It has been shown that the observed high-energy neutrino flux can be described equally well by a combination of a dark matter component and a diffuse astrophysical flux with a power–law energy spectrum. However, neither analysis identified a significant dark matter excess in the data, and models in which the cosmic neutrinos flux arises entirely from dark matter decay are disfavoured.

From the non-observation of a dark matter signal, lower limits are set on the lifetime of dark matter particles with mass above $10^{4}\,\hbox {GeV}$. For such heavy particles these limits are presently the strongest on the dark matter lifetime (see Fig. 8).
